# Hormone-mediated growth dynamics of the barley pericarp as revealed by magnetic resonance imaging and transcript profiling

**DOI:** 10.1093/jxb/erv397

**Published:** 2015-08-14

**Authors:** Rainer Pielot, Stefan Kohl, Bertram Manz, Twan Rutten, Diana Weier, Danuše Tarkowská, Jakub Rolčík, Miroslav Strnad, Frank Volke, Hans Weber, Winfriede Weschke

**Affiliations:** ^1^Leibniz-Institut für Pflanzengenetik und Kulturpflanzenforschung, D-06466 Gatersleben, Germany; ^2^Fraunhofer Institut für Biomedizinische Technik (IBMT) Simulation, Visualization & Magnetic Resonance, Ensheimer Str. 48, D-66386 St. Ingbert, Germany; ^3^Laboratory of Growth Regulators, Centre of the Region Haná for Biotechnological and Agricultural Research, Institute of Experimental Botany ASCR and Palacký University, Šlechtitelů 11, CZ-78371, Olomouc, Czech Republic

**Keywords:** Auxin, barley pericarp, cell expansion, directed growth, gibberellic acid, grain length, growth dynamics, magnetic resonance imaging, transcript profiling.

## Abstract

The shape of the maternal pericarp affects cereal grain mass and yield. Pericarp growth was analysed by magnetic resonance imaging (MRI), revealing topological maps of mobile water in developing pericarp of barley (*Hordeum vulgare*) and displaying tissue regions actively elongating in specific temporal–spatial patterns. Correlation analysis of MRI signals and growth rates reveals that growth in length is mediated by dorsal and also lateral rather than ventral regions. Growth in thickness is related to ventral regions. Switching from dorsal to ventral growth is associated with differential expression of axial regulators of the *HD-ZipIII* and *Kanadi/Ettin* types, and *NPH3* photoreceptors, suggesting light-mediated auxin re-distribution. Auxin increases with the highest levels in the basal pericarp at 6 days after fertilization (DAF), together with transcriptionally up-regulated auxin transport and signalling. Gibberellin biosynthesis is transcriptionally up-regulated only later, and levels of bioactive gibberellins increase from 7 to 13 DAF, with higher levels in ventral than dorsal regions. Differential gene expression related to cell expansion indicates genes related to apoplast acidification, wall relaxation, sugar cleavage, water transport, and cell wall biosynthesis. Candidate genes potentially involved in pericarp extension are distinguished by their temporal expression, representing potential isoforms responsible for dorsal-mediated early growth in length or ventral-mediated late growth in thickness.

## Highlight

Magnetic resonance imaging provides an understanding of dynamics of barley pericarp growth and development, while transcript and hormone profiling unravels a role for auxin and gibberellins in spatial–temporal regulation.

## Introduction

The pericarp of barley grains originates from the gynoecium and represents a major part of the fruit. In the early grain, it consists of the outer epidermis, several layers of parenchyma cells, a three-cell-layered chlorenchyma, and the inner epidermis (Cochrane and Duffus, 1980). The main adaxial bundle of the developing grain extends longitudinally along the crease to the distal end of the placenta–chalazal zone. The developing pericarp is a very heterogeneous tissue. Starch accumulates around the two lateral vascular bundles and, later on, also adjacent to the upper region of the nucellar projection (Weschke *et al.*, 2000). This starch becomes remobilized, and programmed cell death (PCD) is initiated in certain areas such as style regions and around vascular bundles (Radchuk *et al.*, 2009). The function of the persisting chlorenchyma is poorly understood, but could provide oxygen by photosynthesis to support respiration (Rolletschek *et al.*, 2004). During late development, remnants of the pericarp become part of the protective hull.

The final shape and size of the barley caryopsis depends on the temporal and spatial regulation of cell division and cell expansion, and is determined by growth characteristics of the early pericarp. Since cell division in the pericarp is terminated as early as 2 days after fertilization (DAF), its further growth depends predominantly on cell expansion. During the pre-storage phase, an increase in fresh mass of the caryopsis occurs by elongation of the pericarp predominantly in the longitudinal direction, and grain length increases >5-fold until 10 DAF (Radchuk *et al.*, 2011). Grain length is best correlated with final grain mass and volume, and the main events defining final grain mass occur during the first third of the grain-filling period when the pericarp undergoes considerable expansion (Lizana *et al.*, 2010). Thus, growth and development of the early pericarp are important yield-related parameters controlling final grain size and shape.

Cell elongation involves a sequence of multiple steps such as cell wall relaxation to accommodate water uptake, mechanosensing and cell wall extension by turgor pressure, dehydration/cell wall stiffening, and cell wall biosynthesis (Kalve *et al.*, 2014). Plant cell expansion is tightly regulated, and auxin is one of the most important signals (Perrot-Rechenmann, 2010; Kutschera and Wang, 2015). Auxin activates H^+^-ATPases, which acidify the apoplast and activate cell wall proteins such as expansins and xyloglucan endotransglycosylases/hydrolases (XTHs) mediating cell wall loosening. H^+^-ATPases also induce hyperpolarization of plasma membranes, which induces K^+^ inward-rectifying channels essential for water uptake (Fuchs *et al.*, 2006). Accumulation of osmotic active solutes is caused by sucrose catabolism via vacuolar invertases, which leads to higher turgor pressure, subsequent water influx, and cell wall extension (Kutschera and Niklas, 2013). Auxin also induces gene expression of plasma membrane H^+^-ATPase, K^+^ channels, expansins, and cell wall-remodelling enzymes, and promotes export of new cell wall material. Unidirectional auxin transport between different cells and tissues is most important for driving organ shape and pattern formation, which depends on the localization of influx (AUX1/LAX) and efflux carriers (PIN/ABCB) at the plasma membrane (Zažímalová *et al.*, 2010). In developing *Arabidopsis* embryos, PIN1 maintains basipetal polar auxin fluxes along the apical–basal embryonic axis and thereby directs pattern shaping (Friml *et al.*, 2003). AUX/LAX proteins generate auxin sinks and auxin fluxes for lateral root development (Marchant *et al.*, 2002). In Arabidopsis, genes regulating auxin synthesis and transport are expressed in the gynoecium, and transcription factors essential for gynoecium and fruit patterning are directly related to auxin dynamics (Ferrandiz *et al.*, 1999).

Phototropic stimuli can generate asymmetric distribution of auxin, which then causes differential growth on the two sides of a plant organ (Sakai and Haga, 2012). Photoreceptors such as the NPH3 orthologue in rice, *coleoptile phototropism1* (*cpt1*), induce asymmetric auxin redistribution in coleoptiles during the phototropic response (Haga *et al.*, 2005). Cell elongation can also be stimulated by gibberellins (GAs), which are frequently detected in young seeds (Yamaguchi, 2008). In *Arabidopsis*, *de novo* GA biosynthesis after fertilization promotes the initial elongation of siliques (Hu *et al.*, 2008). Auxin acts synergistally to GA, and auxin signals originating in the seed up-regulate GA biosynthesis and signalling in ovules and valves, thereby stimulating fruit growth (Seymour *et al.*, 2013).

Shaping of plant tissues is achieved through changes in the ratio of water to cell material (Ivakov and Persson, 2013). Magnetic resonance imaging [MRI; ^1^H-nuclear magnetic resonance (NMR)] creates topological representations of mobile water fractions as well as mobile (restricted motions) ^1^H-containing biomolecules and can be used to examine plant organ structure, developmental changes, growth characteristics, and metabolic activity. The changes in the abundance and binding state of water are related to changes in physiological characteristics such as tissue structure, growth and development, sugar content, and metabolism (Robinson *et al.*, 2000). MRI has been used to analyse the physiological alterations between plant tissues during fruit maturation (Ishida *et al.*, 1994; Joyce *et al.*, 2002), flower bud development (Yooyongwech *et al.*, 2008), storage root growth (Snaar and Van As, 1992; Metzner *et al.*, 2014), and seed germination (Manz *et al.*, 2005; Rathjen *et al.*, 2009). Generally, high and low signal intensities can be assigned to a high concentration of water in the vacuoles and cytoplasm, respectively (Van As, 2007). High MRI signal intensities denote a high density of hydrogen nuclei, high local molecular mobility, and high abundance of free water (Stark *et al.*, 2007). Hence, MRI signals in highly vacuolated cells in maize shoot apices are related to the presence of free water (Yooyongwech *et al.*, 2008). Signal intensity is often positively correlated with cell and/or vacuolar size (van der Weerd *et al.*, 2001). Increasing signals in the mid parenchyma rings of sugar beet indicate increased vacuole size following cell expansion (Metzner *et al.*, 2014).

The aim of this work is to acquire an understanding of the dynamics of pericarp growth and development and of the molecular factors involved. Since the tissue organization of the pericarp is heterogeneous and varies between the different regions, MRI was used to analyse this organ during development in order to identify cell regions which are responsible for critical growth periods and thus for defining grain shape and size. These studies were combined with the analysis of differential gene expression and the measurement of the distribution of auxin and GAs along the developing pericarp.

## Materials and methods

### Plant hrowth and harvest

Barley (*Hordeum vulgare* L. cv. Barke) was grown in greenhouses with 16h light and 8h darkness. Stages of grain development were determined as described (Weschke *et al.*, 2000). Grains were collected between 10:00 and 12:00h at 2 d or 4 d intervals following anthesis until 14 DAF, and pericarp was manually separated from endosperm and used for RNA analysis.

### Histological analysis

Whole caryopses at 4 DAF and 10 DAF were chemically fixed with 2% glutaraldehyde and 2% formaldehyde in cacodylate buffer (50mM, pH 7.0, 16h). Samples were washed in buffer and water (20min), and dehydrated in a graded ethanol series following by embedding in Spurr’s low viscosity resin. Semi-thin (2 μm) sections were made on a Reichert-Jung Ultracut S (Leica, Vienna, Austria), stained with crystal violet, and examined with a Zeiss Axioimager light microscope (Carl Zeiss, Jena, Germany).

### RNA isolation, labelling, and array hybridization

For transcriptome analysis, an Agilent 8×60K customized barley array was used (Kohl *et al.*, 2015). The design is available at EMBL-EBI ArrayExpress, accession E-MTAB-3040. Total RNA was extracted from freshly isolated pericarps with a Spectrum™ Plant Total RNA Kit (Sigma Aldrich, Steinheim, Germany) and integrity was confirmed using Bioanalyser (Agilent Technologies). A 100ng aliquot of RNA was used for cRNA synthesis and Cy3 labelling via a Low Input Quick Amp Labeling Kit (Agilent Technologies). Labelling efficiency, amount, and quality of cRNA were assured using an ND-1000 Spectrophotometer (NanoDrop Technologies, Wilmington, DE, USA) and Bioanalyser. A 600ng aliquot of labelled cRNA was used for fragmentation and array loading (Gene Expression Hybridisation Kit, Agilent Technologies). Hybridization was carried out for 17h at 65 °C. Arrays were scanned at 5 μm resolution (Agilent Technologies Scanner G2505C) and images were evaluated (determination of spot intensities, background correction) with Feature Extraction V11.5 (Agilent Technologies).

### Data evaluation

Data evaluation was done with Genespring V12.5 (Agilent Technologies). Values were log_2_ transformed and quantile normalized before relative expression values were calculated by subtracting the median expression of each probe from other values of this specific probe (baseline transformation). After removing outliers and transcripts without significant expression at any time point, ANOVA [*P*≤ 0.005, fold change (FC) ≥3] and false discovery rate (FDR) correction (Benjamini–Hochberg) was performed. To identify transcripts with similar expression profiles, K-means clustering (Pearson correlation, 30 clusters) was performed. In order to reduce the number of total clusters, the median values of each cluster were subjected to hierarchical clustering (Pearson correlation), and 10 new clusters were derived.

### Normalization of data sets

For a statistical comparison of the grey value distribution, the NMR data sets of all developmental stages were normalized using the Imaging Software ImageJ (http://rsb.info.nih.gov/ij/) and the PlugIn ‘Stack Normalizer’. This PlugIn recalculates the grey values of the stack by normalization to the minimum grey value of 0 and to the maximum grey value of 255 in each data set.

### 4-D warping and analysis of displacements

Image warping is a process of transforming one (source) image to a second (target) image by redefinition of the spatial relationships between the pixels. It can be based on matching of certain image features such as important points (landmark-based) or on matching of local grey value (or colour) distributions (intensity-based). Intensity-based warping was used to combine normalized 3-D *in vivo* MRI images of successive developmental stages into a virtual 4-D model visualizing the growing caryopsis (Supplementary Video S1, longitudinal direction; Supplementary Video S2, cross direction; Supplementary Video S3, saggital direction, available at *JXB* online). The iterative warping process was based on conversion of grey value data sets into gradient data sets and definition of a displacement vector field, which determined the spatial correspondence and drove the subsequent volume warping. The warped data set was saved and then fed into the next iteration cycle and warped again until the 15th iteration step.

4-D warping is a suitable tool to visualize morphological changes of developing grains across time; details and a comparison with a landmark-based warping approach are described in Pielot *et al.* (2008); for further information about warping, see Toga and Thompson (1999).

The analysis of displacements was performed with the ImageJ-PlugIn PIV (Particle Image Velocimetry) (https://sites.google.com/site/qingzongtseng/piv). For the analysis, slices in all three spatial directions were cut out of the original 3D-NMR data sets (source image) and the warped data sets (after the sixth warping step: target image). The source image and the target image are divided into subimages, and the cross-correlation between the corresponding subimages measures the local optic flow. For longitudinal slices, subimages with the size 8×8 pixels and for the transverse slices subimages with the size 4×4 pixels were used. The correspondence between subimages is defined by the same spatial location of the subimages in the source and the target image, and the optic flow in this study shows the pattern of growth from the younger stage (source image) to the older stage (target image). The direction and magnitude of the optic flow (the displacement) are depicted by a colour-coded displacement vector.

After the analysis, the colour-coded displacement vectors were overlaid with the source image, and the vectors around the embryo region were removed for visualization of the growth processes in the pericarp. The scale bar in Fig. 5 denotes the range of the magnitudes of the vector. 4-D analyses of morphological changes visualize caryopsis growth along the different axes.

### MRI

The MRI experiments were carried out at the Fraunhofer-Institute of Biomedical Engineering, St. Ingbert, Germany, using a Bruker Avance 400 NMR spectrometer (Bruker, Rheinstetten, Germany) with a vertical 9.4 T magnet and a proton resonance frequency of 400 MHz. The micro-imaging equipment consisted of a Micro 2.5 gradient coil yielding a maximum gradient of 0.95 T m^–1^.

The grains were carefully placed inside a horizontal 5mm rf coil. All images were acquired with a standard 3-D spin-echo imaging sequence. The resolution range was set to 4mm and to 4mm or 2mm along the axial and transverse directions, respectively, with 512×256×256 points acquired along each direction. Image resolution was ~31 μm in the axial and ~16 μm in the transversal direction. The echo and repetition times were set to 4–5ms and 250–300ms, respectively. Depending on the grain size and number of points acquired, the total acquisition time for a 3-D image was typically ~5–6h up to 11h for four accumulations (highest quality) in this measurement set.

### Quantitative analysis of auxin and GAs

Samples were analysed for GA content as described (Urbanova *et al.*, 2013) with some modifications. Seed samples (30mg of fresh mass) were homogenized in 2ml polypropylene tubes with 1ml of 80% (v/v) acetonitrile containing 5% (v/v) formic acid and 19 internal GA standards ([^2^H_2_]GA_1_, [^2^H_2_]GA_3_, [^2^H_2_]GA_4_, [^2^H_2_]GA_5_, [^2^H_2_]GA_6_, [^2^H_2_]GA_7_, [^2^H_2_]GA_8_, [^2^H_2_]GA_9_, [^2^H_2_]GA_12_, [^2^H_2_]GA_12_ald, [^2^H_2_]GA_15_, [^2^H_2_]GA_19_, [^2^H_2_]GA_20_, [^2^H_2_]GA_24_, [^2^H_2_]GA_29_, [^2^H_2_]GA_34_, [^2^H_2_]GA_44_, [^2^H_2_]GA_51_, and [^2^H_2_]GA_53_) (OlChemIm, Olomouc, Czech Republic) using an MM 301 bead mill (Retsch, www.retsch.com) at a frequency of 27 Hz for 3min after adding 2mm zirconium oxide beads to each tube to increase the extraction efficiency. The tubes were then placed in a 4 °C fridge and extracted overnight with constant stirring at a frequency of 15rpm using a laboratory Stuart SB3 rotator (Bibby Scientific Ltd, Staffordshire,UK). The homogenates were then centrifuged for 10min at 4 °C (19 000rpm, 10min, 4 °C; Beckman Avanti™ 30). Supernatants were further purified using mixed mode anion exchange cartridges (Waters, www.waters.com) and analysed by ultra-HPLC (Acquity UPLC™ System; Waters) coupled to a triple-stage quadrupole mass spectrometer (Xevo^®^ TQ MS, Waters) equipped with an electrospray ionization (ESI) interface. Gases were detected using multiple-reaction monitoring mode (MRM) based on transition of the precursor ion [M-H]^–^ to the appropriate product ion. Data were acquired and processed by Masslynx 4.1 software (Waters), and GA levels were calculated on the basis of the standard isotope dilution method.

The auxin analysis was performed as described earlier by Pěnčík *et al.* (2009) with some modifications. Briefly, frozen barley seed samples (10mg of fresh mass) were homogenized in liquid nitrogen with a pestle and mortar, and extracted for 5min with 1ml of cold phosphate buffer (50mM; pH 7.0) containing 0.02% sodium diethyldithiocarbamate and [^2^H_5_]indole-3-acetic acid (IAA) as an internal standard. After centrifugation (36 000 *g*; 10min; +4 °C), each sample was transferred into an Eppendorf tube, acidified with 1M HCl to pH 2.7, subjected to a C8-based solid-phase extraction, methylated with ethereal diazomethane, and subsequently purified by immunoaffinity extraction. The final analysis was done by ultra-HPLC (Acquity UPLC™ System; Waters) coupled to tandem mass spectrometer (Xevo^®^ TQ MS, Waters) equipped with an ESI interface operating in positive mode. Data were acquired and processed by Masslynx 4.1 software (Waters), and IAA levels were calculated using the standard isotope dilution method on the basis of auxin detection in MRM mode.

## Results

### Caryopsis length is determined by pericarp growth

**Fig. 1.**
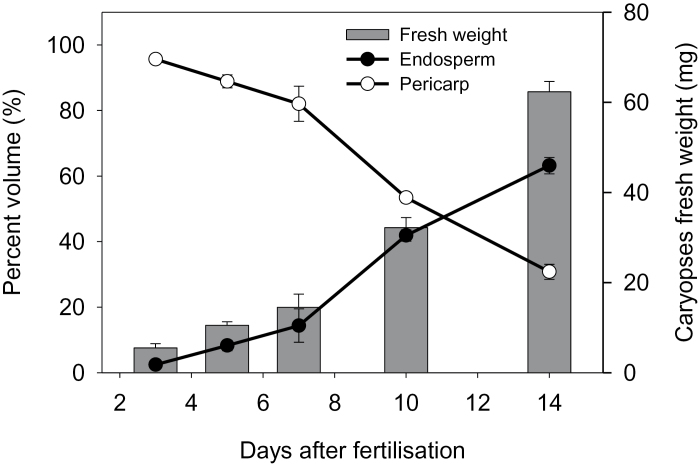
Growth characteristics of barley pericarp and endosperm during development. Bars, fresh mass; curves, percentages of relative volumes. Values are means (*n*=5) ±SD.

**Fig. 2.**
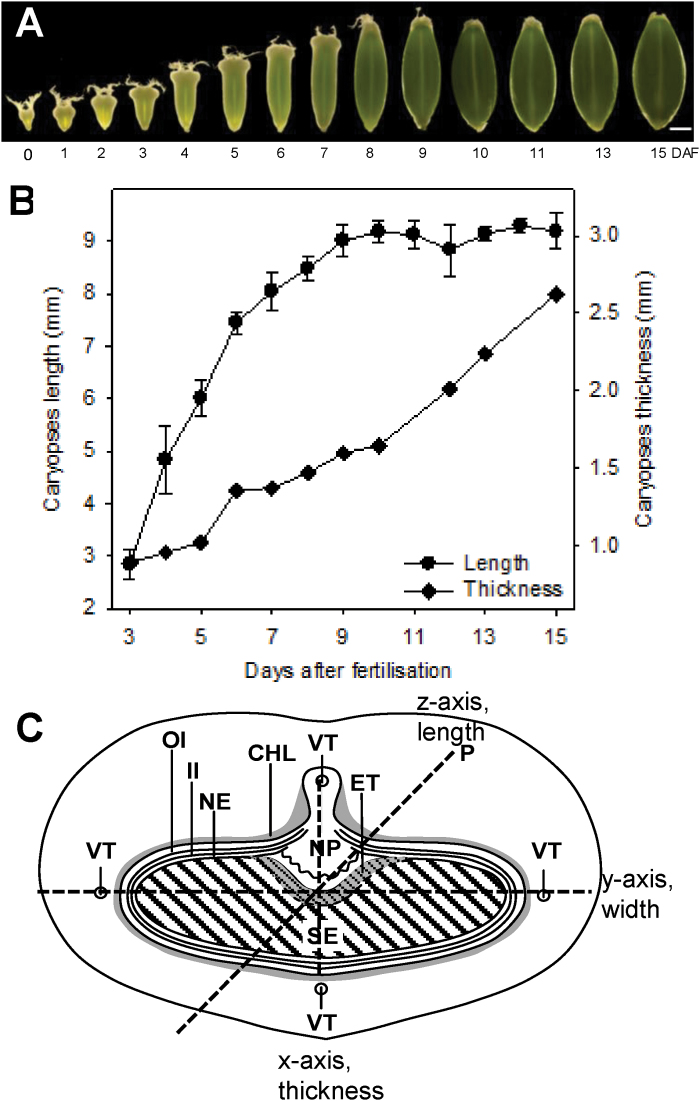
Growth characteristics of barley grain during development. (A) Photographs taken of barley caryopses between anthesis and 14 DAF at 1 d intervals. Scale bar=2mm. (B) Caryopsis grain dimensions in the *x*- and *z*-axe. (C) Schematic transverse section through a caryopsis at 7 DAF highlighting the dimension by *x*-, y-, and *z*-axes, Chl, chlorenchyma; ET, endosperm transfer cells; II, inner integument; NE, nucellar epidermis; OI, outer integument; P, pericarp; SE, starchy endosperm; VT, vascular tissue. (This figure is available in colour at *JXB* online.)

During early development, the grain volume is predominantly determined by the pericarp. At ~10 DAF, the percentages of pericarp and endosperm volumes are equal (Fig. 1). From this stage onwards, the grain volume strongly increases due to endosperm growth, and grain fresh mass accumulates linearly (Weschke *et al.*, 2000). Between anthesis and 9 DAF, the caryopsis predominantly grows in the longitudinal orientation (*z*-axis) and only slightly in thickness (*x*-axis) (Fig. 2A, B). After 10 DAF, growth in thickness (*x*-axis) dominates. A schematic overview of the spatial tissue organization in the developing caryopsis is shown in Fig. 2C.

The results show that growth of the early caryopsis in length and to a minor extent in thickness is ruled by the maternal pericarp. Because cell proliferation in the pericarp is completed by 2 DAF (Radchuk *et al.*, 2011), the increase in length is due to cell elongation.

### High and low ^1^H-NMR signal intensities in the developing pericarp are related to persisting and disintegrating cell regions, respectively

**Fig. 3.**
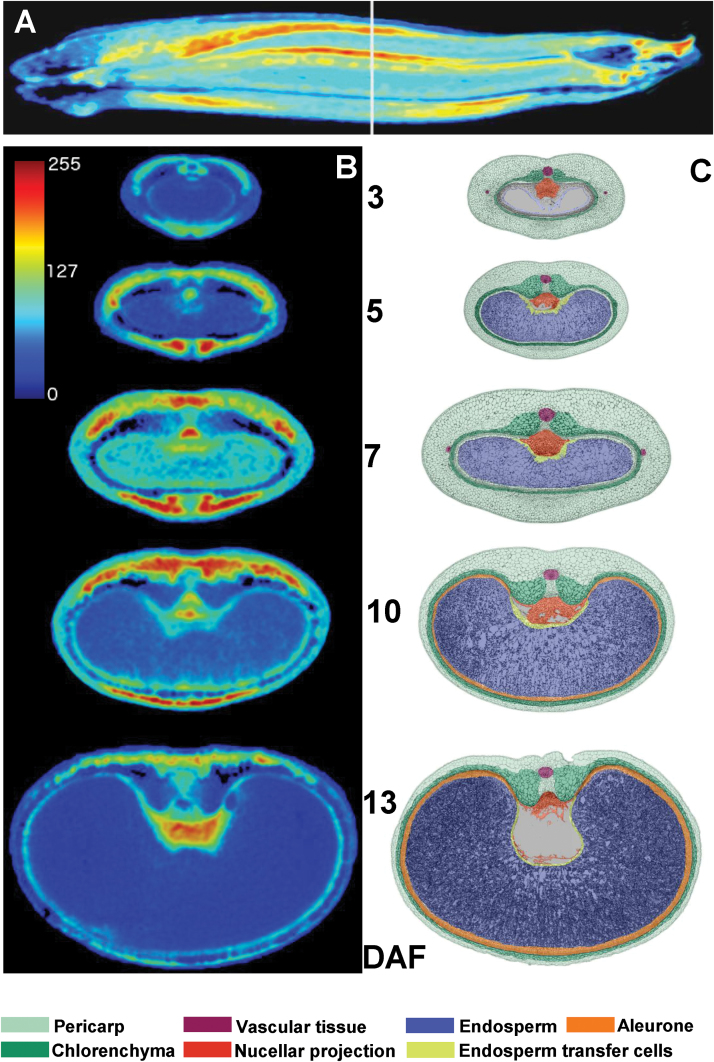
MRI signal intensities. (A) Distribution of MRI signal intensities in a 7 DAF caryopsis. (B) Median–transverse slices at 3, 5, 7, 10, and 13 DAF. Signal intensities are presented in a colour code, scale bar=1mm. (C) Morphological structures and schematic overview showing the tissue organization of the developing caryopsis.

**Fig. 4.**
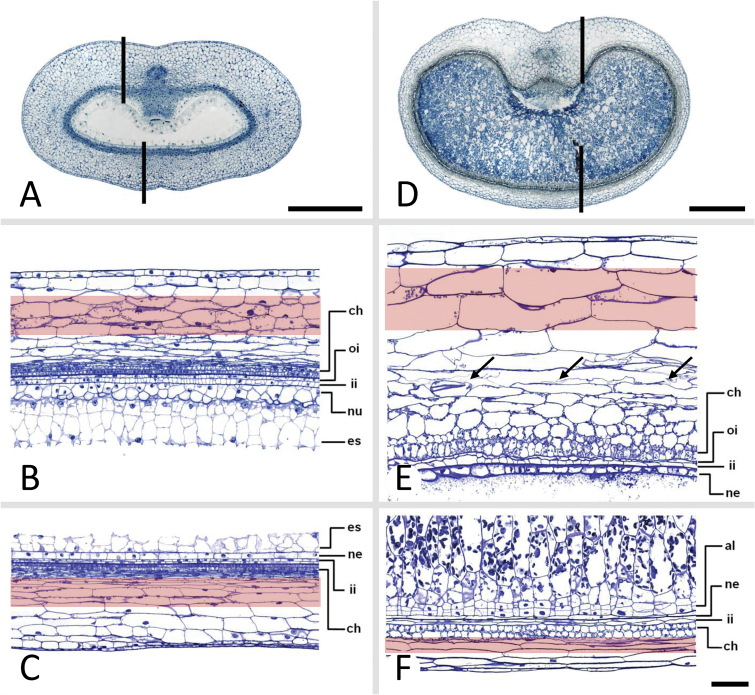
Transverse (A, D) and longitudinal (B, C, E, F) histological sections of caryopses at 4 DAF (A–C) and 10 DAF (D–F). Arrows in (E) denote regions with particularly low MRI signals showing signs of deterioration. Al, aleurone; ch, chlorenchyma; es, endosperm; ii, inner integument; ne, nucellar epidermis; oi, outer integument; scale bar=400 μm (A, D), 100 μm (B, C, E, F).

MRI signal intensity was analysed in freshly harvested grains at 3, 3.5, 4, 5, 6, 7, 8, 9, 10, 11, 12, 13, and 15 DAF. Figure 3A shows a colour-coded MRI intensity map of a whole caryopsis at 7 DAF. The median–transverse MRI slices of caryopses at 3, 5, 7, 10, and 13 DAF are given in Fig. 3B. To identify the position of the median slice, grain length was estimated from the MRI images to select the slice which represents 50% of the grain length (indicated in Fig. 3A). In the intensity maps, red colour denotes high proton density and/or high local molecular mobility, and blue represents low proton density and/or low local molecular mobility. MRI intensity maps of whole caryopses of all developmental stages and median–transverse and median–longitudinal MRI slices of each individual caryopsis are presented in colour code and grey scale in Supplementary Fig. S1 (whole caryopses) and Supplementary Fig. S2 (median slices) at *JXB* online. Since images are based on normalized MRI signals, colour-coded intensities are comparable between all stages. Developmental alterations were visualized for patterns of signal intensities based on median–transverse slices (Fig. 3B). The corresponding fine morphological structures and a schematic overview showing the tissue organization of the developing caryopsis are presented in Fig. 3C. Signals of highest intensity (red colour) were found in the pericarp between 5 and 10 DAF, the nucellar projection at 7 DAF, and the endosperm cavity at 13 DAF (Fig 3B). Between 10 and 14 DAF, increasing signal intensities in the endosperm cavity were accompanied by a linear increase of grain fresh mass (Fig. 1). Signals were generally low in the endosperm, except for the moderate levels present in the whole endosperm at 7 DAF and in regions of the differentiating aleurone at 10 and 13 DAF (Fig. 3B). At all stages, signal intensities were higher within distinct pericarp regions compared with endosperm, except for the endosperm cavity at 13 DAF. Signals were especially high in areas surrounding and flanking the dorsal minor vascular bundle at 5 and 7 DAF and in the ventral region, especially at 10 DAF (Fig. 3B). These regions with high MRI signals were analysed histologically based on transverse sections through mid-caryopses at 4 (Fig. 4A) and 10 DAF (Fig. 4D), with the positions of the longitudinal section planes (*z*-axis) indicated. At 4 DAF, cells in the ventral region underlying high MRI signals (red-shaded area; Fig. 4B) are moderately elongated, whereas those in the dorsal region (Fig. 4C) are extremely drawn-out in the longitudinal (*z*-axis) direction. At 10 DAF, ventral cells (Fig. 4E) have been elongated in both the *z*- and *x*-directions, whereas those in the dorsal part retained their long drawn-out shape in the *z*-direction (Fig. 4F). Arrows in Fig. 4E denotes regions with particularly low MRI signals, showing signs of deterioration, possibly by PCD.

The results show that high MRI signals display a specific axis-symmetric, temporal–spatial pattern, namely within specific regions of the dorsal, ventral, and lateral pericarp, arranged along the dorsal–ventral axis and extending through the ventral crease region and the dorsal minor vascular bundle. Obviously, these regions denote localized growth in the pericarp.

### 4-D analyses of morphological changes visualize caryopsis growth along the different axes

**Fig. 5.**
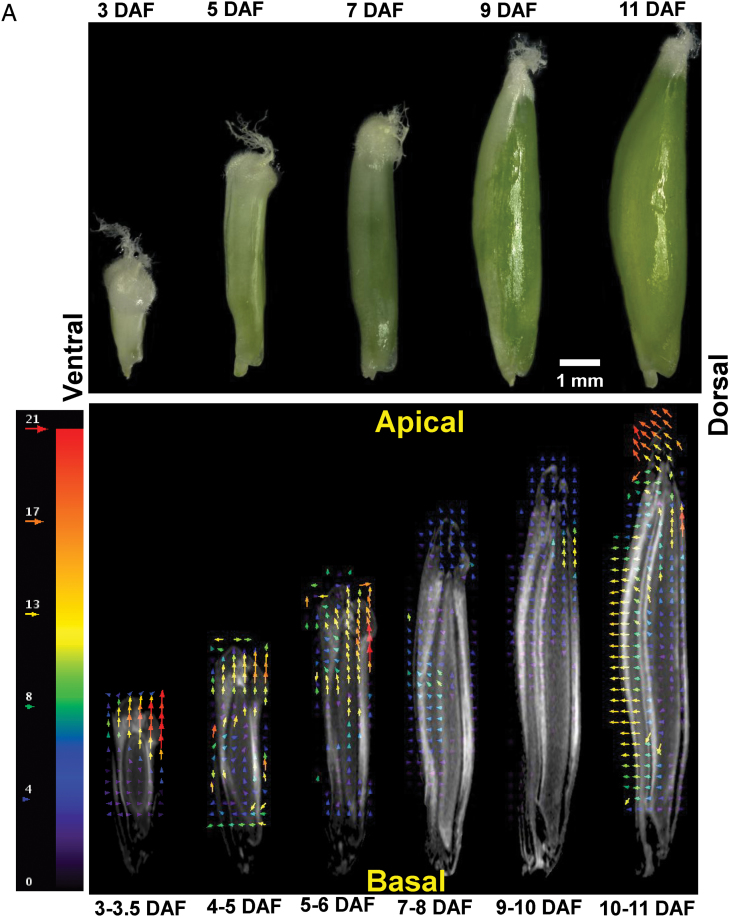

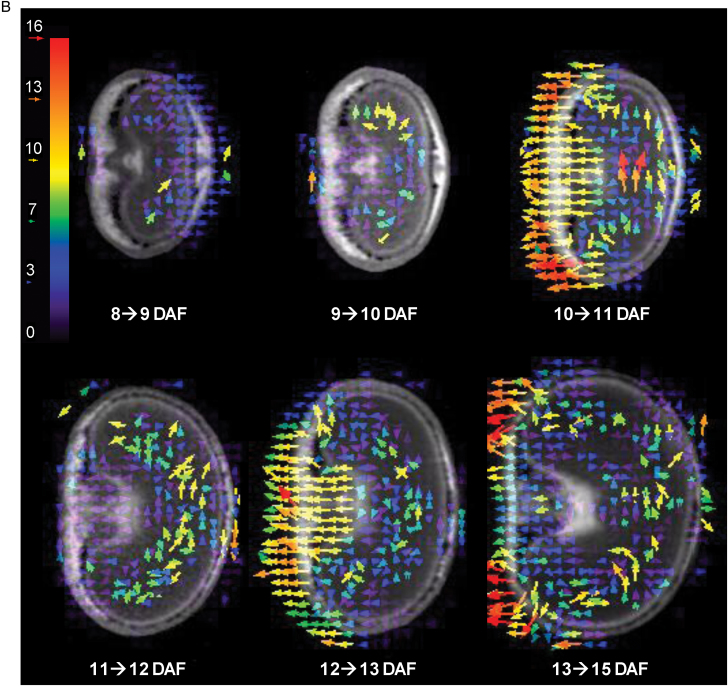
Morphological changes of developing caryopses with time. (A) Photographs showing extension of caryopses along the *z*-axis between 3 and 11 DAF (upper panel) and distribution of displacement vectors outlined for six developmental stages along the *z*-axis of the caryopses (lower panel). (B) Distribution of displacement vectors on transverse slices (*x*- and *y*-axes); the colour code of vectors reflects the scale from low (blue) to high displacement (red).

The distribution of displacement vectors is outlined for six developmental stages for longitudinal slices (*z*-axis) from 3.5 to 11 DAF (Fig. 5A) and for transverse slices (*y*- and *x*-axes) from 8 to 15 DAF (Fig. 5B). The colour code of the vectors reflects the scale from low (blue) to high displacement or growth rates (red). From 3.5 to 6 DAF, vectors along the *z*-axis are up to 5-fold more intense, especially within the dorsal pericarp region, compared with those on the *x*-axis. At 7/8 DAF, vectors on both *z*- and *x*-axis are similar but lower in intensity. At 9/10 DAF, vector intensity in the longitudinal (*z*-axis) direction increased again, especially within the dorsal pericarp, whereas those in the transverse (*x*-axis) direction remained low. At 10/11 DAF, vector intensity increased to higher values for both longitudinal and transversal directions (Fig. 5A). Vector distribution shown on the transverse slices reveals low intensities from 8 to 11 DAF. At 10/11 DAF, high intensity vectors are frequent in the ventral and especially in the lateral pericarp and in the adjacent endosperm along the *x*-axis. At 11/12 DAF, vector intensity decreases particularly in the pericarp, but not in the endosperm region. At 12/13 DAF, high intensity vectors are present in the ventral pericarp and the adjacent endosperm, with lower values in the lateral pericarp. At 13/15 DAF, the intensity slightly decreases in the ventral pericarp but increases in the lateral pericarp (Fig. 5B).

The 4-D warping data indicate that morphological changes occur in a defined temporal–spatial pattern. Stages from 3 to 6 DAF are dominated by expansion in length in the longitudinal *z*-axis, and are most probably driven by unidirectional elongation of the dorsal pericarp regions adjacent to the style, while there is only low expansion in the transverse direction (*x*- and *y*-axis) at that stage. The period between 7 and 10 DAF, which designates the transition phase, reveals only minor changes in caryopsis dimensions. From 11 to 14 DAF, expansion dominates in the *x*-axis, which obviously is mainly driven by the ventral pericarp. At that stage, the dorsal pericarp is apparently no longer involved in determining grain shape, which is reflected by low MRI signals in the dorsal pericarp (Figs 3B, 4B) and strongly reduced expansion rates in the longitudinal direction (Fig. 2B).

### High MRI signal intensities in dorsal and lateral pericarp regions are correlated with the longitudinal growth rate of the caryopsis

**Fig. 6.**
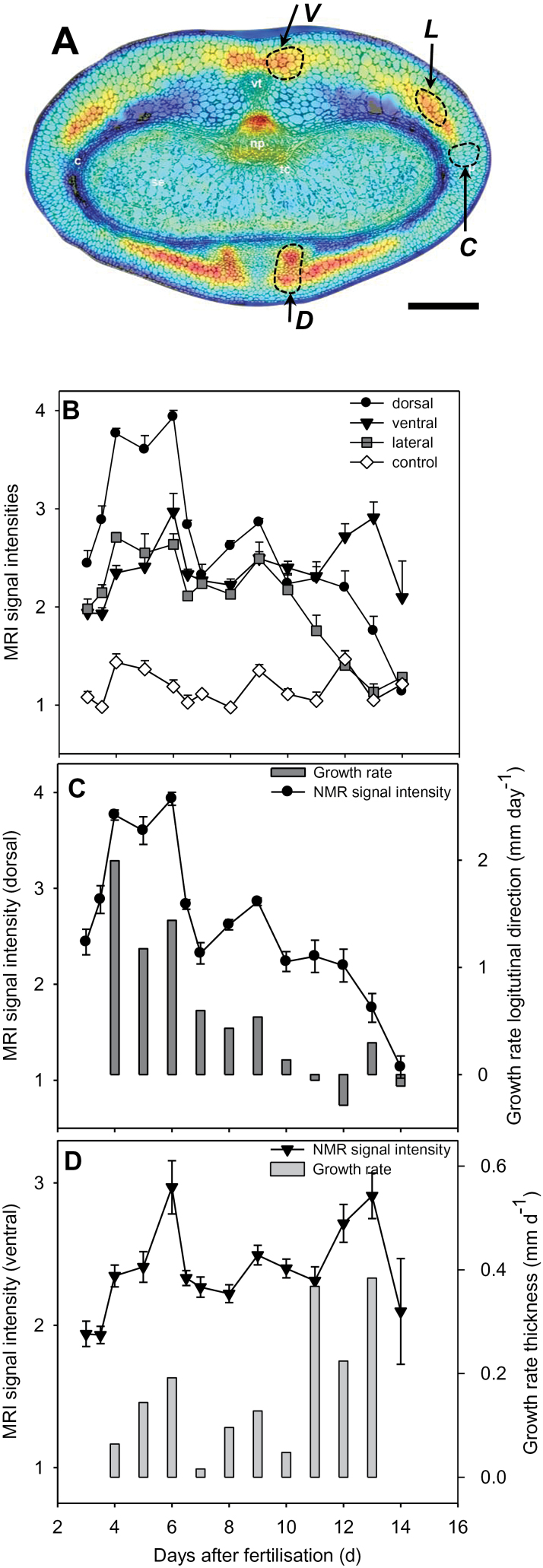
MRI signal intensities and growth rates of caryopses. (A) Distribution of MRI signal intensities in the median transversal slice of a caryopsis at 7 DAF, scale bar = 0.5mm. (B) Profiles of MRI signal intensities in the dorsal, lateral, ventral, and control regions. (C) Profile of the MRI signal intensity in the dorsal region and growth rate in the longitudinal (*z*-axis) direction. (D) Profile of MRI signal intensity in the ventral region and growth rate in the transverse (*x*-axis) direction. (This figure is available in colour at *JXB* online.)

To analyse whether MRI signals are associated with pericarp growth characteristics, intensities were quantified from median transverse slices in dorsal (D in Fig. 6A), ventral (V), and lateral regions (L). Signals adjacent to the lateral minor vascular bundle (Fig. 6A, region C) were used as controls. The highest signals occurred in dorsal regions (D) between 4 and 6 DAF (Fig. 6B) and at 9 DAF. Thereafter, intensities decreased continuously to control levels until 15 DAF. The pattern of MRI signals in the lateral region (L) was highly correlated with that of region D (Spearman correlation, *r* ^2^=0.87, *P*<0.001), but intensities were significantly lower at all stages, except at 7, 10, and 15 DAF. The control profiles (C) were independent of those of D, V, and L. In the ventral region, signal intensities peaked at 6 and 13 DAF and decreased thereafter. The intensity curve was not correlated with those of either regions D or L, and is thus independent.

Pericarp growth rates for length and width were calculated from the changing grain dimensions (Fig. 2B) and plotted together with signal intensity profiles in dorsal and ventral regions (Fig. 6C, D). The longitudinal growth rat coincided and was highly correlated with MRI signal intensities in the dorsal (Spearman correlation, *r* ^2^=0.88, *P*<0.001) and the lateral region (Spearman correlation, *r* ^2^=0.87, *P*<0.001), with the highest values between 4 and 6 DAF. No correlation existed between longitudinal growth rate and the signal intensity curve in the ventral region (Spearman correlation, *r* ^2^=0.01, *P*=0.8). Growth rates for thickness were highest between 11 and 13 DAF. Whereas at 6 and 9 DAF high MRI signals were concurrently present in dorsal, lateral, and ventral regions, the signal at 13 DAF is restricted only to the ventral region (Fig. 6B). The growth rate for thickness was moderately correlated with the signal intensity curve in the ventral region (Spearman correlation, *r* ^2^=0.56, *P*=0.07) but not with that in the dorsal and lateral region (Spearman correlation, *r* ^2^=0.24, *P*=0.48).

The results indicate that MRI signal intensities in the dorsal and lateral regions were linked to each other, and together were highly correlated to the longitudinal pericarp growth rate. On the other hand, the MRI signal intensities in the ventral region do not show a clear correlation, but eventually could be associated with the caryopses growth rate for thickness.

### Transcript profiling of the developing pericarp

The temporal arrangement of areas of anisotropic growth in the pericarp should be reflected at the level of transcriptional activities. Using a custom-made 8×60-K barley microarray (Kohl *et al.*, 2015), transcript profiling of the pericarp was performed at anthesis (0 DAF), and at 4, 8, 10, and 14 DAF, yielding 7374 transcripts with at least 2-fold significant differences between at least two stages (Supplementary Table S1 at *JXB* online).

**Fig. 7.**
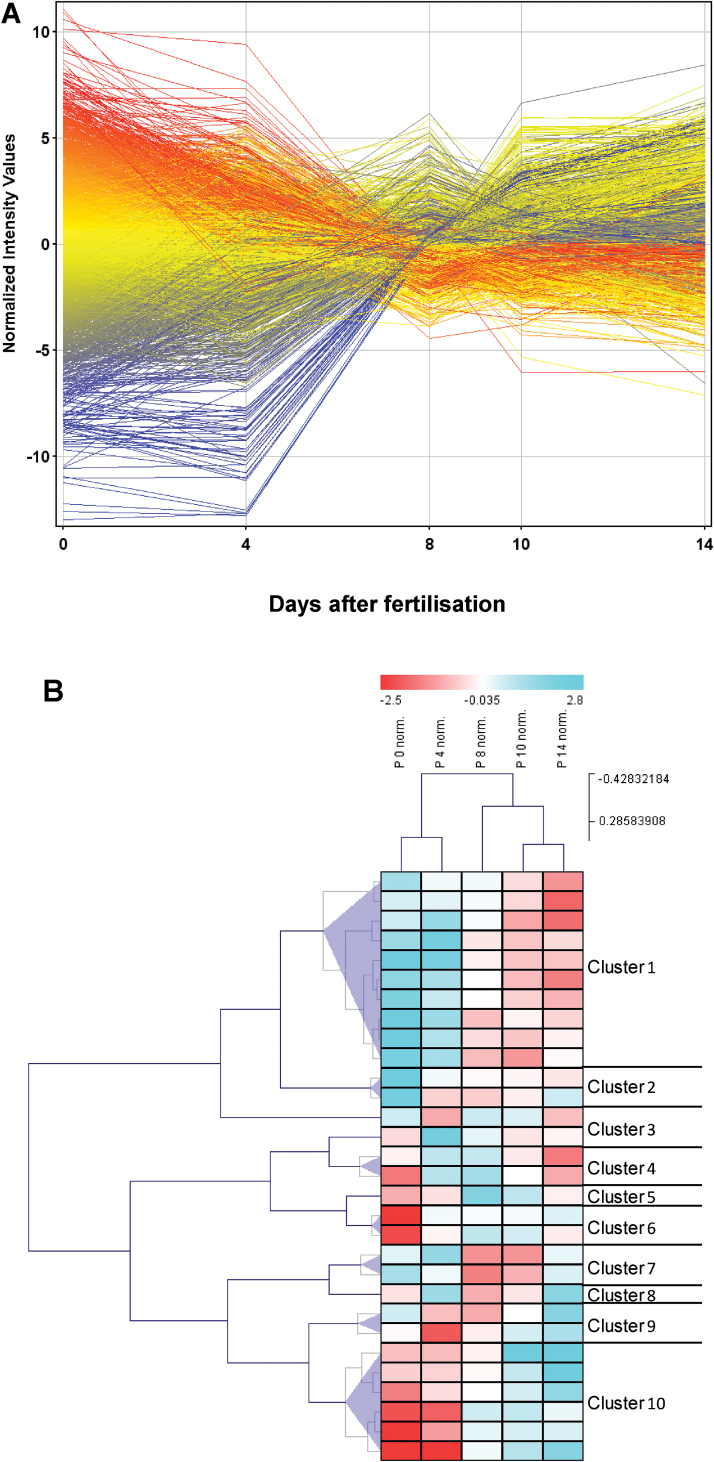
Expression profiles and cluster analysis of differentially expressed genes. (A) Profiles of 7374 transcripts during pericarp development derived from microarray experiments (Agilent 8×60K customised barley array); each time point represents three biological replicates. Raw expression values were log2-transformed, quantile normalized, and centred. Differential expression was detected by ANOVA (*P*<0.005, FC>3); single profiles were coloured according to values at 0 DAF. (B) Cluster analysis: K-means clustering (Pearson correlation) with 30 clusters was performed and median expression values are colour-coded from dark blue (high relative expression) to dark red (low relative expression). To reduce the total cluster number, the median expression profiles were subjected to hierarchical clustering and summarized to nine new clusters according to hierarchical grouping (clusters 1, 2, and 4–10), with cluster 3 containing outliers.

General expression profiles (Fig. 7A) showed a clear distinction between transcripts highly expressed at 0 and 4 DAF and at 10 and 14 DAF, while DAF 8 marked a crossover point. This separation into three phases was confirmed by hierarchical clustering of median expression values derived from K-means clustering within samples (Fig. 7B, upper panel). A total of 4475 (64.4%) transcripts were assigned to functional categories (BIN-codes, Supplementary Table S2 at *JXB* online) using Mercator (Thimm *et al.*, 2004) and, to identify enriched categories within specific expression profiles, the data set was subjected to hierarchical clustering and divided into 10 main expression profiles. Among genes preferentially expressed early (0–4 DAF, clusters 1 and 2), enriched categories are associated with cell growth and development (DNA, 86% of all genes in this BIN; cell functions, 62%; cell wall, 62%; RNA, 60%; nucleotide, 55%; protein, 48%) and with primary metabolism (glycolysis, 58%; carbohydrate metabolism, 56%; amino acid metabolism, 44%). Enriched categories of genes preferentially expressed late (10– 14 DAF, cluster 10) are related to mitochondrial electron transport/ATP synthesis (52%), stress (50%), secondary metabolism (41%), and transport (38%). Hormone-related gene expression is moderately enriched in both early (33%) and late pericarp (31%). A breakdown of the category ‘hormone function’ (Supplementary Table S3) revealed that genes related to auxin and GA functions (52.5%) are most abundant in the early pericarp, whereas genes related to auxin and GA (40%), ethylene (19%), and jasmonic acid (17%) are abundant in the late pericarp. High percentages of auxin- and GA-related genes in clusters 1, 2, and 10 point to auxin- and/or GA-mediated growth in the early and late pericarp.

### Gene expression pattern related to auxin- and gibberellin-mediated growth

Auxin rapidly stimulates cell elongation in most plant organs, and triggers early developmental events such as directed growth and shape patterning. The functions of auxin depend on its local concentration. Specific influx and efflux transporters can relocate auxin within organs and generate local sinks, which provokes pattern formation and directed cell elongation (Ljung, 2013).

**Fig. 8.**
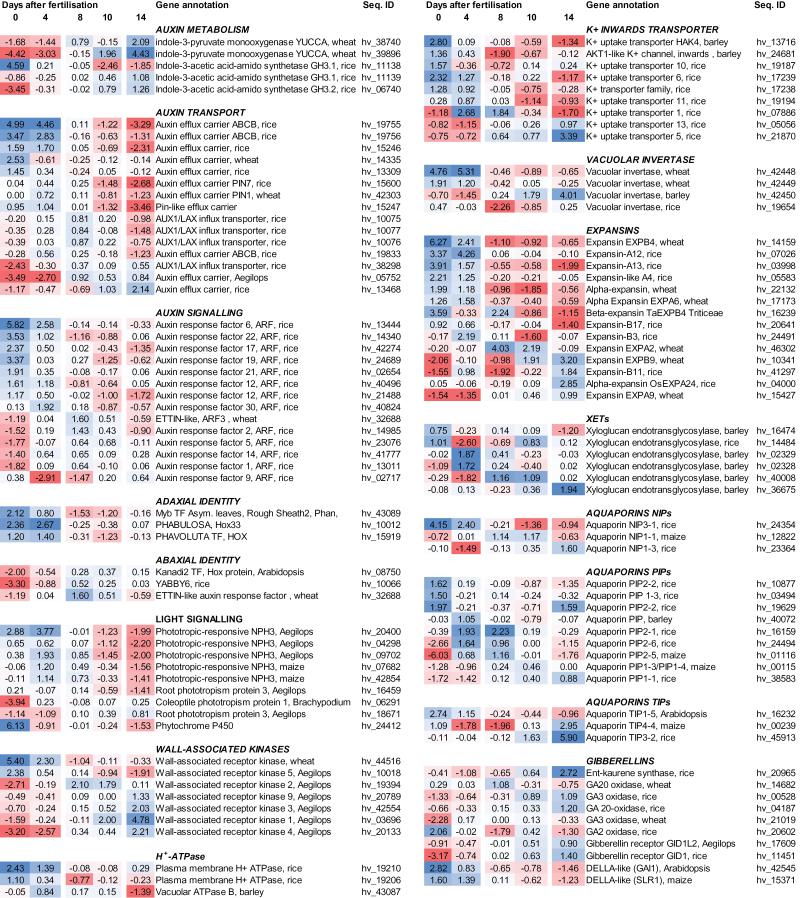
Expression profiles of genes associated with auxin and GA functions and cell expansion. Colours indicate the level of gene expression from high (dark blue) to low (dark red).

Only two genes involved in auxin metabolism were highly expressed in the pericarp (Fig. 8); both isoforms of the biosynthesis enzyme indole-3-pyruvate monooxygenase were expressed most highly at 14 DAF. Isoforms of indole-3-acetic acid-amido synthetases, involved in auxin inactivation, were highly expressed either in early (0 DAF) or late stages (14 DAF). Unlike auxin metabolism, transport was represented by differential expression of as many as 15 genes during pericarp growth. Eight efflux transporters of the PIN and ABCB families were most highly expressed at 0–4 DAF whereas members of the AUX1/LAX influx carrier family were expressed preferentially at 8–10 DAF. Two efflux transporters were most highly expressed at 14 DAF (Fig. 8).

Auxin triggers growth by a complex signalling system (Hayashi, 2012). Transcriptional regulation by auxin response factors (ARFs) is important to activate downstream genes. In the pericarp, 14 ARFs displayed differential gene expression; among them, eight were maximally expressed at 0 and 4 DAF. Five ARFs were most highly expressed at 8 and 10 DAF, and only one member at 14 DAF. Specific patterns of gene expression indicated that during early development auxin transport and signalling prevailed, rather than auxin biosynthesis.

As well as auxins, GAs were also frequently involved in cell expansion. Ten sequences related to GA metabolism, homeostasis, and signalling were differentially expressed. Regarding GA biosynthesis, *ent*-kaurene synthase, GA 20-oxidase (GA20ox) and GA3ox were expressed at the highest extent at 14 DAF, together with two sequences encoding the GA receptor GID1. On the other hand, GA2ox involved in GA inactivation and two negative regulators of GA action, the DELLA proteins GAI1 and SLR1, were highly expressed at 0 DAF. The observed differential expression of GA-related genes indicated that, unlike auxin, GA obviously is more important for pericarp expansion during later development.

### Gene expression indicates a switch from adaxial to abaxial activity

Photoreception can induce asymmetric auxin distribution in hypocotyls, coleoptiles, and roots dependent on light response (Sakai and Haga, 2012). In rice, phototropic-responsive NPH3 orthologues mediate auxin redistribution in coleoptiles (Haga *et al.*, 2005). In the pericarp, eight NPH3-like members were differentially expressed. Five showed maxima at 4 DAF, two at 0 DAF, and one at 14 DAF. A phytochrome P450-related gene was very strongly expressed only at 0 DAF.

In *Arabidopsis* leaves, adaxial–abaxial patterning is auxin mediated and depends on transcription factor activities promoting either an adaxial or abaxial fate (Scarpella *et al.*, 2010). The HD-Zip III factors *PHABULOSA* and *PHAVOLUTA* are restricted to the adaxial side and promote adaxial cell fate, whereas the Kanadi-like proteins together with ARF3/ETT repress HD-Zip III activities and promote abaxial fate (Pekker *et al.*, 2005). In the barley pericarp, transcription factor genes homologous to the *Arabidopsis* genes *ASYMMETRIC LEAVES* (*AS*), *PHABULOSA* (*PHB*), and *PHAVOLUTA* (*PHV*), which are related to adaxial identity, were preferentially expressed at 0 and 4 DAF, whereas homologues of *Kanadi*, *Ettin*, and *Yabby*, and which are related to abaxial identity, were most highly expressed between 8 and 10 DAF. This indicates a temporal switch of gene expression of homologues conferring adaxial to those effecting abaxial patterning during pericarp growth.

### Gene expression pattern related to cell expansion-mediated growth

Auxins are closely related to cell elongation, and differentially expressed genes related to expansion-mediated growth are listed in Fig. 8. Auxin-inducible H^+^-ATPases acidify the apoplast, which activates voltage-dependent K^+^ channels. The resulting K^+^ uptake facilitates water influx to promote cell expansion (Ringli, 2010). In the pericarp, two plasma membrane and one vacuolar H^+^-ATPase were preferentially expressed at 0–4 DAF. Of nine differentially expressed K^+^ inwards transporters, five revealed the highest expression at 0 DAF, two at 4 DAF, and another two at 14 DAF. Decreased apoplastic pH, as generated by H^+^-ATPases, could activate cell wall-loosening enzymes such as expansins and xyloglucan XETs, which loosen bonds between cellulose and hemicellulose fibrils (Cosgrove, 2000). From the six XETs, gene expression of two members peaked at 0 DAF, of two others at 4 DAF, and of two at 8/14 DAF. Four expansins were most highly expressed at 0 and 4 DAF, one at 8 DAF, and another four at 14 DAF. Auxin-mediated turgor-driven enlargement of cells can be stimulated by sucrose cleavage via vacuolar acid invertases, yielding hexoses, which increases the osmotic concentration in vacuoles and facilitates water uptake (Kutschera and Niklas, 2013). During pericarp development, four vacuolar invertases were differentially expressed; three had expression maxima at 0–4 DAF and another one at 10–14 DAF. Aquaporins facilitate the transport of water and small molecules. Water transport is important for cell elongation and osmoregulation (Maurel *et al.*, 2009). During pericarp development, three members encoding nodulin-26-like intrinsic proteins (NIPs) were differentially expressed, with maxima at early, middle, and later stages. One member of the tonoplast intrinsic proteins (TIPs) was most highly expressed at 0 DAF and another two at 14 DAF. Nine isoforms of plasma membrane intrinsic proteins (PIPs) were differentially expressed in the pericarp.

### Spatial–temporal pattern of auxin and gibberellin dynamics during pericarp growth

**Fig. 9.**
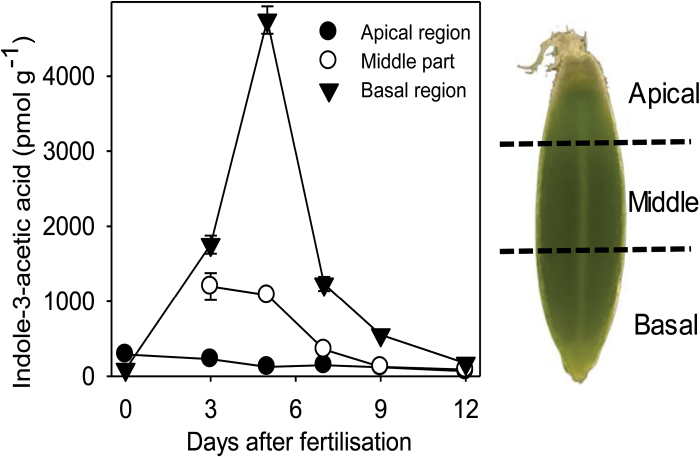
Indole-3-acetic acid (IAA) concentration in basal, middle, and apical pars of the barley caryopsis between 0 and 12 DAF. Values are means ±SD, *n* = 3. (This figure is available in colour at *JXB* online.)

Gene expression analysis revealed that both auxins and GAs could be involved in localized growth regulation in different tissues and developmental stages of the pericarp. Therefore, auxin levels were measured in the developing pericarp separated into apical, middle, and basal regions (Fig. 9). At 0 DAF, levels of IAA were generally low but significantly higher in the apical part compared with the basal part (*t*-test, *P*<0.01). However, from 3 to 12 DAF, IAA contents were significantly higher in basal parts compared with middle and apical parts. At 3, 5, 7, and 12 DAF, levels were also significantly higher in middle compared with apical parts. IAA concentrations showed a pronounced profile during development, with a strongly increasing level from 0 to 5 DAF by a factor of 50 (basal part). Thereafter, IAA levels decreased in all parts until 12 DAF. The distribution pattern of IAA in the pericarp showed the highest levels between 3 and 7 DAF, coinciding with the highest growth rates for length. Except for 0 DAF, the values indicated a longitudinal IAA gradient with decreasing concentrations from basal to apical regions.

**Fig. 10.**
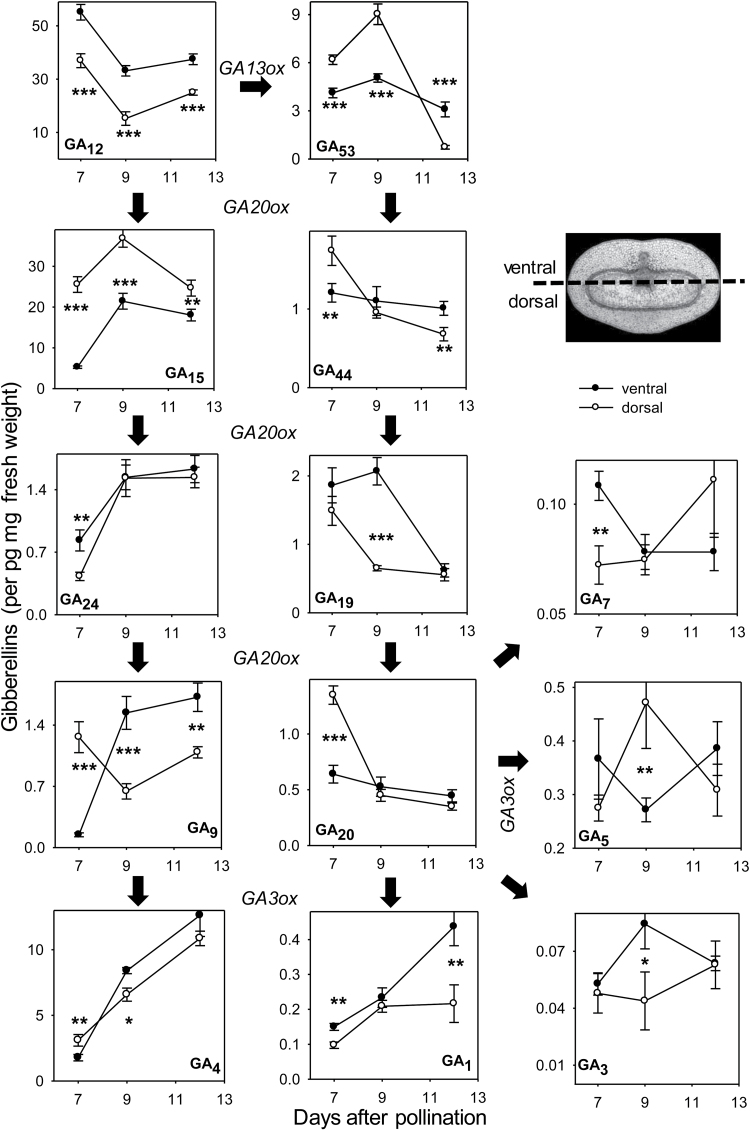
Gibberellin concentrations in dorsal and ventral parts of the caryopsis. Simplified GA biosynthesis pathway and concentrations of 13 GA metabolites determined between 3 and 12 DAF. Data are means ±SD, *n* = 4; significant differences according to Student’s *t*-test, *P<0.05, **P<0.02, ***P<0.001.

GA levels were measured in isolated pericarp fractions divided into ventral and dorsal parts. Thirteen different GAs identified in the pericarp are shown in Fig. 10, along with a simplified pathway of their biosynthesis including bioactive compounds GA_1_, GA_3_, GA_4_, GA_5_, and GA_7_ derived from precursors GA_12_ and GA_53_. GA_12_ and GA_53_ were oxidized in three to four steps in parallel pathways into GA_9_ and GA_20_ by GA20oxs, the 2-oxoglutarate-dependent dioxygenases (2ODDs). Formation of bioactive compounds was catalysed by a GA3ox, another 2ODD (Hedden and Phillips, 2000; Yamaguchi, 2008). In pericarp, concentrations of the 13-non-hydroxylated GAs GA_12_, GA_15_, GA_24_, and GA_9_, and their bioactive biosynthetic product GA_4_ were relatively higher compared with the parallel pathway of 13-hydroxylated GAs starting from GA_53_. Among bioactive GAs, GA_4_ was ~20-fold more abundant than the second most highly abundant GA_1_ and >30- and 100-fold more abundant compared with GA_5_ and GA_3/7_. The levels of the two most abundant bioactive GAs, GA_4_ and GA_1_, increased in both dorsal and ventral parts from 7 to 13 DAF by a factor of 4–5. The GA_4_ level was lower in the ventral part at 7 DAF but higher at 9 and 13 DAF (not significant at 13 DAF). The GA_1_ level was higher in the ventral parts at all three stages (not significant at 9 DAF).

The results indicated that in early pericarp the 13-non-hydroxylated pathway of GA biosynthesis is preferred, leading to the most abundant bioactive GA_4_, levels of which, together with that of GA_1_, increase from 7 to 13 DAF. Moreover, their concentrations are relatively higher in the ventral compared with the dorsal parts.

## Discussion

The shape of the maternal pericarp affects cereal grain mass by constraining grain expansion and, consequently, grain volume (Ugarte *et al.*, 2007). Therefore, knowledge of the growth characteristics of the pericarp is highly significant to understand grain yield formation because grain length is the best correlated trait with final grain mass (Lizana *et al.*, 2010). Since the pericarp is very heterogeneous, MRI was used to identify those regions which are critical for growth and thus determine grain shape and size. It was found that pericarp growth is a dynamic and sequential process, starting with elongation in the longitudinal direction until the grain reaches its final length at 9 DAF, followed by increasing thickness in the transverse direction. Transcript and hormone profiling unravels a potential role for auxin and GAs in spatial–temporal regulation of pericarp growth.

### MRI reveals physiological changes during pericarp development

The barley pericarp grows mainly by cell expansion because cell division terminates very early after fertilization (Radchuk *et al.*, 2011). Plant cell expansion and organ shaping are derived from the changing relationship of water and cell wall material (Burton and Fincher, 2014). MRI is a suitable method to uncover physiological changes during organ development by measuring the ^1^H-NMR signals, which provide topological maps of mobile water. Signal intensities are correlated with vacuolar size and assigned to high concentrations of free water (Robinson *et al.*, 2000; Van As, 2007; Metzner *et al.*, 2014). Since water uptake is the driving force for plant cell elongation, the distribution of MRI signal intensities in the pericarp produces maps of actively elongating regions. The distribution of MRI signals reveals specific axis-symmetric, temporal–spatial patterns, arranged along the dorsal–ventral axis, extending longitudinally through the ventral crease region and the dorsal minor vascular bundle. Intense MRI signals are restricted to certain areas of the pericarp and appear first in dorsal and lateral regions at 5 DAF and later in ventral areas at 11 DAF (Figs 3, 6A). Thus, patterns of high MRI signals reveal active pericarp growth only in distinct regions, which change over time. On the other hand, low signal activity designates differentiated cells, which accumulate starch, surrounding the lateral vascular bundles (Weschke *et al.*, 2000). Low signals are also assigned to weakly stained and potentially degrading cells with large intercellular spaces (Fig. 4E, arrows), which possibly are undergoing PCD (Radchuk *et al.*, 2011).

Quantified MRI signals show that the distribution patterns in dorsal and lateral regions are highly correlated with each other but are different from those in the ventral area. Moreover, the pattern of the longitudinal growth rate is correlated with MRI signal intensities in dorsal and lateral rather than ventral regions. It is concluded that MRI signals in the dorsal and lateral regions together denote pericarp growth in length rather than in thickness. Significant expansion occurred later only in the transversal direction (*x*-axis) (Fig. 2B). Temporal coincidence of growth rates and MRI signals suggests that growth in thickness is more strongly guided by cells in the ventral region adjacent to the main vascular bundle and occurs when elongation in the *z*-axis is terminated.

4-D warping of morphological changes visualizes the distribution of displacement vectors along the different axes over time and therefore reveals the temporal–spatial and oscillating pattern of caryopsis growth. The period from 4 to 6 DAF is dominated by expansion in length, driven by unidirectional elongation of dorsal pericarp regions. The period from 11 to 14 DAF is dominated by expansion in width/thickness, driven by expansion of the ventral pericarp (Fig. 5). Hence, the 4-D warping results confirm those from the correlation analysis between growth rate and MRI signal intensities.

A fundamental difference in growth between dorsal and ventral regions is also reflected in the behaviour of cell expansion (Fig. 4). A recent quantification reveals that cells in the dorsal pericarp only increase in length—by 230%—but not in width, between 1 and 10 DAF, whereas the ventral cells increase 228% and 65% in length and width, respectively (Radchuk *et al.*, 2011). Hence, dorsal cells become long, narrow, and cylindrical, whereas the ventral cells expand in both longitudinal and transverse directions. Histological analysis (Fig. 4) confirms the different cell expansion characteristics of ventral cells (*z*- and *x*-axis) and dorsal cells (*z*-axis) underlying the regions of high MRI signals. This confirms the hypothesis that the dorsal cell mediates growth in length and ventral cells also in thickness.

### Length extension of the pericarp may be mediated by unidirectional auxin import

The temporal arrangement of localized, anisotropic growth in the pericarp is reflected at the transcript level. Array-based transcript profiling during pericarp development at early stages, 0–4 DAF, reveals up-regulation of functions such as cell growth and development, cell wall, RNA, nucleotide, and protein, and primary metabolism. The later stages, 10–14 DAF, reveal preferential activities related to mitochondrial electron transport/ATP synthesis, stress, secondary metabolism, and transport. Auxin functions are most abundant in the early pericarp, while those related to auxin and GA, ethylene, and jasmonic acid are most abundant in the late pericarp.

Auxin frequently stimulates cell elongation in most plant organs and triggers early development such as directed growth and organ shape patterning. Auxin function often depends on its local concentration generated by influx and efflux transporters relocating auxin. Whereas the PIN-type transporters mediate auxin efflux, the AUX/LAX family members are influx carriers (Zažímalová *et al.*, 2010; Ljung, 2013). Genetic and biochemical evidence shows that the different members of the auxin carrier family are related to developmental programmes in different organs and tissues (Swarup and Peret, 2012). In the pericarp, eight members of the PIN- and ABCB-type potential auxin efflux transporters are up-regulated at 0 and 4 DAF together with eight ARFs (Fig. 8). In contrast, one of the major auxin biosynthesis enzymes, indole-3-pyruvate monooxygenase (Stepanova *et al.*, 2011), is transcriptionally up-regulated in the pericarp only at late stages, 14 DAF.

In summary, specific patterns of gene expression during pericarp development show that auxin transport and signalling, but not metabolism, are frequently present in the young pericarp. It is thus hypothesized that in the early pericarp auxin is not synthesized *de novo* but is imported, and PIN- and ABCB-type efflux transporters possibly generate and maintain specific gradients. The existence of such a hypothetical gradient along the pericarp longitudinal axis is supported by auxin measurements, which reveal decreasing concentrations from basal to apical regions and the highest levels between 3 and 6 DAF (Fig. 9) coinciding with maximal longitudinal growth rates, which is driven predominantly by the dorsal pericarp region. It can thus be speculated that auxin, imported from the mother plant into the basal region, plays an important role in pericarp shaping, especially for control of longitudinal growth at 4–6 DAF.

It is hypothesized that anisotropic growth within the dorsal pericarp could be mediated by unidirectional auxin import rather than *de novo* biosynthesis, leading to decreasing concentrations along the basal to apical axis, with the highest level at 5 DAF. At the moment this hypothesis is based mainly on correlative evidence. It has to be confirmed by localizing specific AUX1/LAX-type influx and PIN/ABCB-type efflux carriers.

### Switch from adaxial to abaxial activity

Asymmetric distribution of auxin often occurs in response to light stimuli, which then causes differential growth within a plant organ (Sakai and Haga, 2012). NPH3-like proteins are key signal transducers of plant phototropism and can induce asymmetric auxin distribution in plant organs such as hypocotyls, coleoptiles, and roots (Holland *et al.*, 2009). Likewise, the NPH3/CPT1-dependent phototropism of coleoptiles is achieved by lateral auxin translocation and subsequent growth redistribution (Haga *et al.*, 2005). In the pericarp, five members of the NPH3-type photoreceptors are preferentially expressed only at early stages, 4 DAF, when the pericarp is growing exclusively by elongation in the longitudinal direction driven by the dorsal region. Thus, preferential growth of the dorsal cells at 4–6 DAF could eventually be accompanied by NPH3 photoreceptor functions involved in auxin re-distribution. Light signalling could be important since the dorsal side of the pericarp is turned towards the outside of the ear and is exposed to light.

Various aspects of plant development are frequently regulated by HD Zip III transcription factors in an auxin-dependent manner (Itoh *et al.*, 2008). *PHB* and *PHV* effect establishment of the shoot apical meristem and adaxial identity of lateral organs. *PHB* and *PHV* operate antagonistically to KANADI-like transcription factors, and both families are involved in establishing spatial organization. Gain- and loss-of-function mutants also show complementary phenotypes (Ilegems *et al.*, 2010). In the pericarp, there is a temporal switch in preferential gene expression of transcription factors conferring adaxial/abaxial identity in *Arabidopsis* leaves and fruits (Pekker *et al.*, 2005; Kalve *et al.*, 2014). HD-Zip III-like *PHB* and *PHV* are preferentially up-regulated at 0–4 DAF, and those related to abaxial identity (*KANADI* and *ETTIN*) at 8–10 DAF (Fig. 8), indicating alternate dorso-ventral activity during pericarp growth. Accordingly, the barley pericarp displays a dorso-ventral growth pattern, starting within dorsal regions by elongation in the longitudinal direction followed by growth in width/thickness mediated by ventral areas. Obviously, switching from dorsal to ventral growth requires up-regulation of axial regulators specifying differential and sequential growth at the two sides of the pericarp. Likewise, in *Arabidopsis*, HD-Zip III proteins promote axial cell elongation whereas KANADI proteins affect auxin transport by inhibiting PIN gene expression (Ilegems *et al.*, 2010).

### GA accumulation in pericarp may be important for growth in thickness

Cell elongation is stimulated by GAs and/or the synergistic interaction of GAs and auxins. Thereby, GAs act downstream of auxins and are important to stimulate fruit growth (Seymour *et al.*, 2013). GAs also confer cell elongation in the nucellar projection of the barley pericarp (Weier *et al.*, 2014). In *Arabidopsis* seeds, *de novo* GA biosynthesis after fertilization requires gene expression of GA20ox and GA3ox, which promote the initial elongation of siliques (Hu *et al.*, 2008). In the barley pericarp, the GA biosynthesis enzymes *ent*-kaurene synthase, GA20ox and GA3ox are up-regulated only later at 14 DAF, together with the GA receptor GID1 (Fig. 7), indicating that GA could be more important for pericarp expansion during later development. The measurement of 13 GA members of the biosynthetic pathway in both dorsal and ventral parts supports this notion. The data show that the 13-non-hydroxylated pathway of GA biosynthesis is preferentially active in the pericarp, leading to the bioactive GA_4_, the most abundant metabolite in the pericarp, whose levels together with that of GA_1_ steadily increase from 7 to 13 DAF. Moreover, the levels of active GAs are relatively higher in ventral compared with dorsal parts (Fig. 10).

Thus, differential gene expression related to auxin and GA functions indicates that unlike auxin, GA obviously is more important for pericarp expansion in the ventral area during later development, in agreement with higher levels of bioactive GAs in this region and stage.

### Gene expression related to cell expansion reflects stage-dependent candidates

Auxin-mediated cell expansion may be attributable to a sequence of apoplast acidification, wall relaxation and loosening, sugar cleavage, water transport and uptake, and cell wall biosynthesis, with auxin activating transcription of relevant genes (Perrot-Rechenmann, 2010). Expression analysis reveals six XTHs and 14 expansins up-regulated in the pericarp (Fig. 8). One of them, *TaEXPA6*, is expressed in young wheat pericarps and is associated with grain expansion (Lizana *et al.*, 2010). Further up-regulation comprises genes related to apoplast acidification (two H^+^-ATPases), K^+^ influx (nine K^+^ transporters), sugar cleavage (four vacuolar invertases), and water fluxes (15 aquaporins) (Fig. 8). Particular members of these gene families involved in the various steps can be clearly distinguished by the time of their preferential expression. Certain isoforms are up-regulated at 0 and 4 DAF or at 10 and 14 DAF, whereas only a few single members are preferentially expressed at 8 DAF (Fig. 7). Their specific temporal up-regulation suggests that these particular candidates most probably represent such isoforms which are responsible either for dorsal-mediated early growth in length (candidates expressed at 0 and 4 DAF) or for ventral-mediated late growth in width (10 and 14 DAF). However, since this is merely correlative evidence from co-expression analysis, a possible cause and relationship of suggested key players has to be analysed in the future.

## Supplementary data

Supplementary data are available at *JXB* online.

Figure S1. Intensity maps of median–longitudinal MRI slices from caryopses at 3, 3.5, 4, 5, 6, 7, 8, 9, 10, 11, 12, 13, and 15 DAF in colour code and grey scale.

Figure S2. Median–transverse MRI slices from caryopses at 3, 3.5, 4, 5, 6, 7, 8, 9, 10, 11, 12, 13, and 15 DAF in colour code and grey scale.

Table S1. List of 7374 differentially expressed genes during pericarp development.

Table S2. Allocation of genes differentially expressed to functional categories (http://mapman.mpimp-golm.mpg.de/).

Table S3. Allocation of differentially genes expressed into categories ‘Hormone-related gene expression’.

Video S1. Virtual 4-D model visualizing the growing caryopsis in the longitudinal direction.

Video S2. Virtual 4-D model visualizing the growing caryopsis in the cross direction.

Video S3. Virtual 4-D model visualizing the growing caryopsis in the saggital direction.

Supplementary Data
