# Supplementary material for: Hormone-mediated growth dynamics of the barley pericarp as revealed by magnetic resonance imaging and transcript profiling
Source: J Exp Bot. 2015 Aug 14;66(21):6927–43. doi: 10.1093/jxb/erv397 (PMC4623697; doi:10.1093/jxb/erv397)
Supplement: Supplementary Data [file supp_66_21_6927__index.html]

Hormone-mediated growth dynamics of the barley pericarp as revealed by magnetic resonance imaging and transcript profiling — Hormone-mediated growth dynamics of the barley pericarp as revealed by magnetic resonance imaging and transcript profiling — Supplementary Data 

# Hormone-mediated growth dynamics of the barley pericarp as revealed by magnetic resonance imaging and transcript profiling

## Supplementary Data

Data files

- Supplementary Data - Supplementary Data
- Supplementary Data - Supplementary Data
- Supplementary Data - Supplementary Data
- Supplementary Data - Supplementary Data
