# Supplementary material for: Hormone-mediated growth dynamics of the barley pericarp as revealed by magnetic resonance imaging and transcript profiling
Source: J Exp Bot. 2015 Aug 14;66(21):6927–43. doi: 10.1093/jxb/erv397 (PMC4623697; doi:10.1093/jxb/erv397)
Supplement: Supplementary Data [file supp_erv397_jexbot149450_file003.pdf]

Table S2

BINCODE	NAME	C 1	C 2	C 3	C 4	C 5	C 6	C 7	C 8	C 9	C 10	Total No. In BINs	Portion of transcripts (%)
1	Photosynthesis	5	1		1			6	112	1	9	**135**	2.8%
2	major CHO metabolism	30	2		1	2	2	4	4		12	**57**	1.2%
3	minor CHO metabolism	9			1	5	2	4	5	4	12	**42**	0.9%
4	glycolysis	11						1	2	1	4	**19**	0.4%
5	fermentation		1			3		1			1	**6**	0.1%
6	gluconeogenesis / glyoxylate cycle										3	**3**	0.1%
7	OPP	3							2			**5**	0.1%
8	TCA / org. acid transformation	6		1			1	1	2	1	8	**20**	0.4%
9	mitochondrial e$^-$ transport/ATP synthesis	9			1	1	1			2	15	**29**	0.6%
10	cell wall	63	15		4	17	6	16	9	3	54	**187**	3.9%
11	lipid metabolism	43	7		4	19	3	13	5	3	50	**147**	3.1%
12	N-metabolism	3						2	1		1	**7**	0.1%
13	amino acid metabolism	30	1		3	2	3	10	3	4	14	**70**	1.5%
14	S-assimilation	1	1									**2**	0.0%
15	metal handling	7			1	3	1	1	1	1	8	**23**	0.5%
16	secondary metabolism	22	4		3	3	2	11	8	5	41	**99**	2.1%
17	hormone metabolism	41	16	1	4	11	2	26	7	12	54	**174**	3.7%
18	Co-factor and vitamine metabolism	6			1				3		2	**12**	0.3%
19	tetrapyrrole synthesis	6						1	6			**13**	0.3%
20	stress	56	14	2	4	12	3	28	6	9	133	**267**	5.6%
21	redox	7	3		2			3	6	2	10	**33**	0.7%
22	polyamine metabolism	2									1	**3**	0.1%
23	nucleotide metabolism	19	3		2	4	1	1		1	7	**38**	0.8%
24	Biodegradation of Xenobiotics	4			1							**5**	0.1%
25	C1-metabolism	4				1	1					**6**	0.1%
27	RNA	319	53	3	3	20	15	25	26	13	146	**623**	13.1%
28	DNA	178	5	1		3	2	3	3		17	**212**	4.5%
29	protein	400	32	6	4	47	16	65	47	21	260	**898**	18.9%
30	signalling	138	17		3	29	6	32	8	6	115	**354**	7.5%
31	cell	89	14	2	1	7	1	9	6	3	34	**166**	3.5%
33	development	46	21		3	7	3	11	5	13	58	**167**	3.5%
34	transport	85	16	2	3	21	8	39	7	16	120	**317**	6.7%
26	misc	147	40	4	7	60	17	64	28	25	214	**606**	12.8%
	**Total number in cluster**	**1798**	**266**	**22**	**57**	**277**	**96**	**377**	**312**	**146**	**1403**	**4745**	
	**Portion of transcripts (%)**	**37.7%**	**5.6%**	**0.5%**	**1.2%**	**5.8%**	**2.0%**	**7.9%**	**6.6%**	**3.1%**	**29.5%**		100.0%
35	not assigned	934	172	15	29	135	54	191	149	95	855	**2629**	
	**Total no. in cluster incl. not assigned**	**2725**	**438**	**37**	**86**	**412**	**150**	**568**	**461**	**241**	**2256**	**7374**	
	**Portion of transcripts (%)**	**37.0%**	**5.9%**	**0.5%**	**1.2%**	**5.6%**	**2.0%**	**7.7%**	**6.3%**	**3.3%**	**30.6%**	**100.0%**	

Table S2. Allocation of differentially expressed genes in functional categories (http://mapman.mpimp-golm.mpg.de/).

Table S3

BIN code	Hormone metabolism	C 1	C 2	C 3	C 4	C 5	C 6	C 7	C 8	C 9	C 10	
**BIN code 17**	**Hormone metabolism**	43	16	1	4	11	2	26	7	12	52	174
**17_1**	**Abscisic acid (ABA)**	**7**	**1**	**0**	**0**	**0**	**0**	**3**	**1**	**4**	**8**	**24**
17_1_1	ABA synthesis-degradation	2	0	0	0	0	0	2	0	3	3	**10**
17_1_2	ABA signal transduction	0	1	0	0	0	0	0	0	1	3	**5**
17_1_3	ABA ind.-regul.-respons.-activ.	5	0	0	0	0	0	1	1	0	2	**9**
**17_2**	**Auxin**	**18**	**4**	**1**	**2**	**2**	**0**	**6**	**1**	**2**	**13**	**49**
17_2_1	Auxin - synthesis-degradation	0	0	0	1	0	0	0	0	0	3	**4**
17_2_2	Auxin - signal transduction	4	1	0	0	0	0	0	0	0	1	**6**
17_2_3	Auxin ind.-regul.-respons.-activ.	14	3	1	1	2	0	6	1	2	9	**39**
**17_3**	**Brassinosteroid (BS)**	**2**	**0**	**0**	**1**	**1**	**0**	**1**	**0**	**0**	**3**	**8**
17_3_1	BS - synthesis-degradation	2	0	0	1	1	0	1	0	0	2	**7**
17_3_2	BS - signal transduction	0	0	0	0	0	0	0	0	0	1	**1**
**17_4**	**Cytokinin (CK)**	**0**	**3**	**0**	**1**	**4**	**2**	**3**	**0**	**0**	**3**	**16**
17_4_1	CK - synthesis-degradation	0	2	0	0	2	0	1	0	0	0	**5**
17_4_2	CK - signal transduction	0	1	0	1	2	2	2	0	0	3	**11**
**17_5**	**Ethylene**	**6**	**3**	**0**	**0**	**1**	**0**	**8**	**0**	**3**	**10**	**31**
17_5_1	Ethylene - synthesis-degradation	5	3	0	0	1	0	8	0	2	10	**26**
17_5_2	Ethylene - signal transduction	0	0	0	0	0	0	0	0	0	1	**1**
17_5_3	Ethylene ind.-regul.-respons.-activ.	1	0	0	0	0	0	0	0	1	2	**4**
**17_6**	**Gibberellin (GA)**	**5**	**4**	**0**	**0**	**2**	**0**	**1**	**0**	**0**	**8**	**20**
17_6_1	GA - synthesis-degradation	3	3	0	0	1	0	0	0	0	8	**14**
17_6_2	GA - signal transduction	0	0	0	0	1	0	0	0	0	0	**1**
17_6_3	GA ind.-regul.-respons.-activ.	3	1	0	0	0	0	1	0	0	0	**5**
17_7	***Jasmonate (JA)***	***3***	***1***	***0***	***0***	***1***	***0***	***3***	***5***	***3***	***9***	***25***
17_7_1	JA - synthesis-degradation	0	1	0	0	1	0	2	5	3	9	**21**
17_7_3	JA ind.-regul.-respons.-activ.	3	0	0	0	0	0	1	0	0	0	**4**
**17_8**	**Salycylic acid (SA)**	**0**	**0**	**0**	**0**	**0**	**0**	**1**	**0**	**0**	**0**	**1**
17_8_1	SA - synthesis-degradation	0	0	0	0	0	0	1	0	0	0	**1**

Table S3. Allocation of differentially expressed genes into categories “Hormone-related gene expression”.

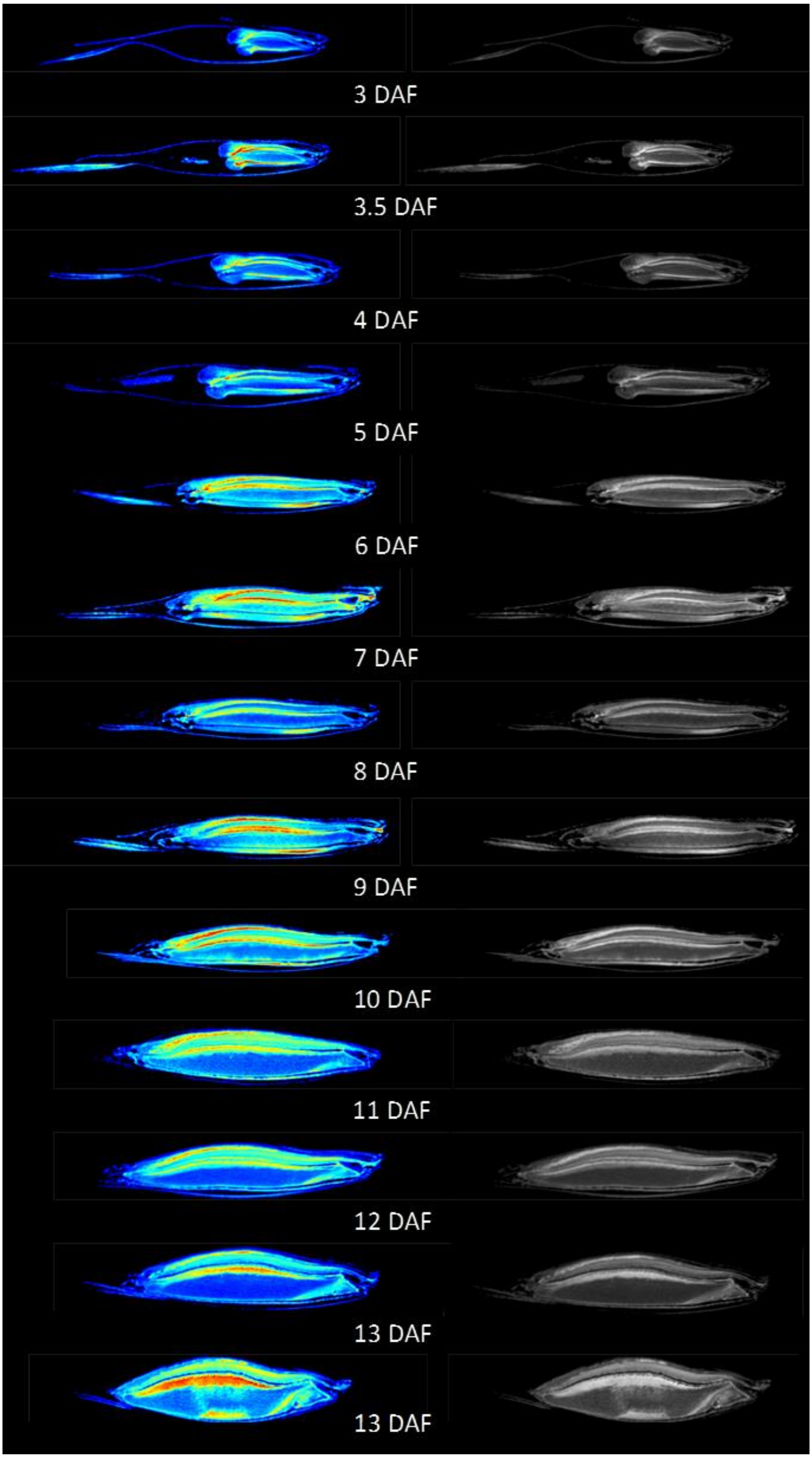

Fig. S1

Figure S1. Intensity maps of median-longitudinal MRI slices from caryopses at 3, 3.5, 4, 5, 6, 7, 8, 9, 10, 11, 12, 13 and 15 DAF in colour code and grey scale. In the colour-coded intensity maps, red colour denotes high proton density and/or high local molecular mobility, blue represents low proton density and/or low local molecular mobility. In the grey-scaled intensity maps, high and low proton density/local molecular mobility is denoted in dark and light grey, respectively.

Fig. S2

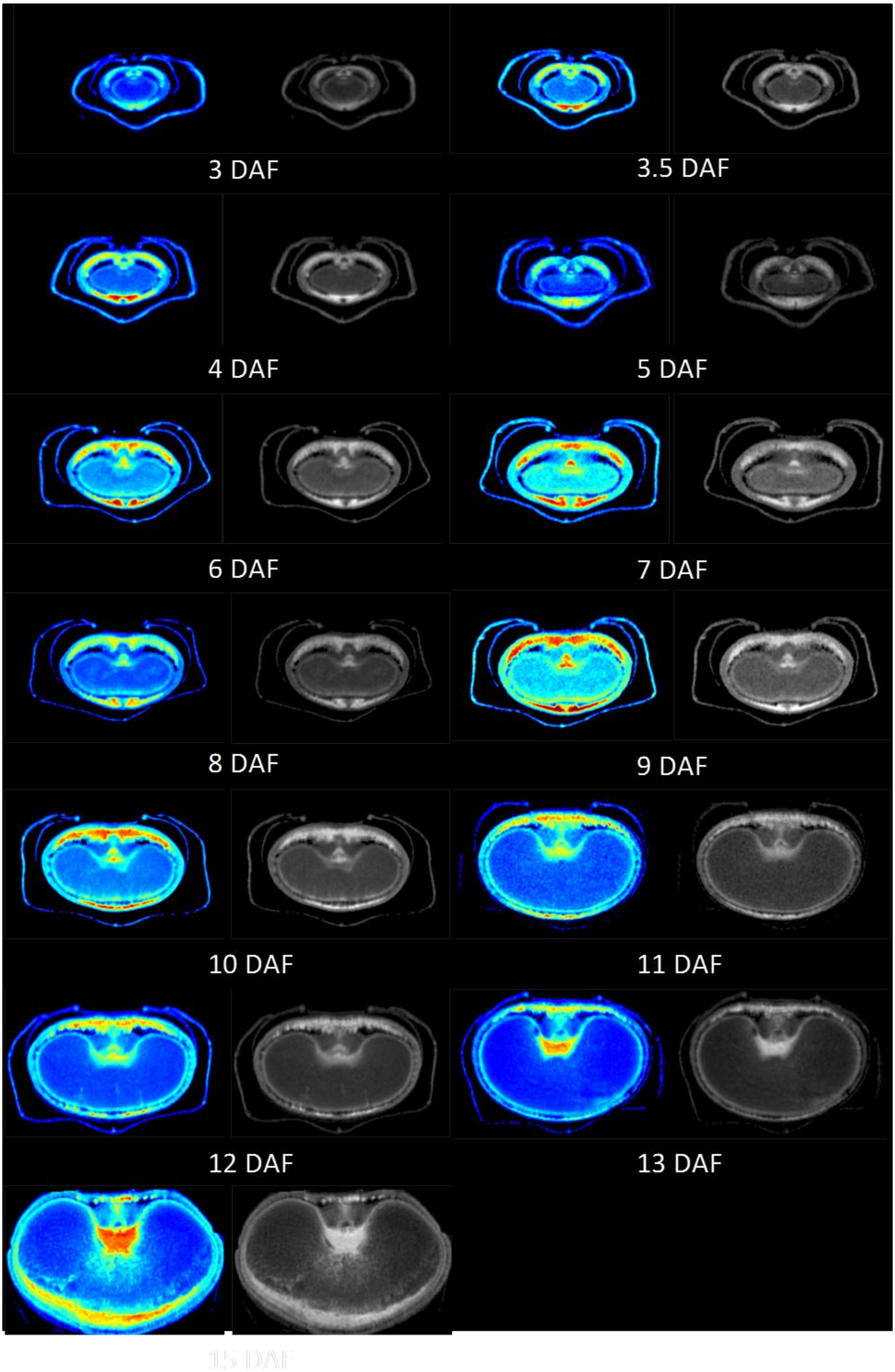

Figure S2. Median-transverse MRI slices from caryopses at 3, 3.5, 4, 5, 6, 7, 8, 9, 10, 11, 12, 13 and 15 DAF in colour code and grey scale.
