# Supplementary material for: Hormone-mediated growth dynamics of the barley pericarp as revealed by magnetic resonance imaging and transcript profiling
Source: J Exp Bot. 2015 Aug 14;66(21):6927–43. doi: 10.1093/jxb/erv397 (PMC4623697; doi:10.1093/jxb/erv397)
Supplement: Supplementary Data [file supp_erv397_jexbot149450_file002.pptx]

## Slide 1
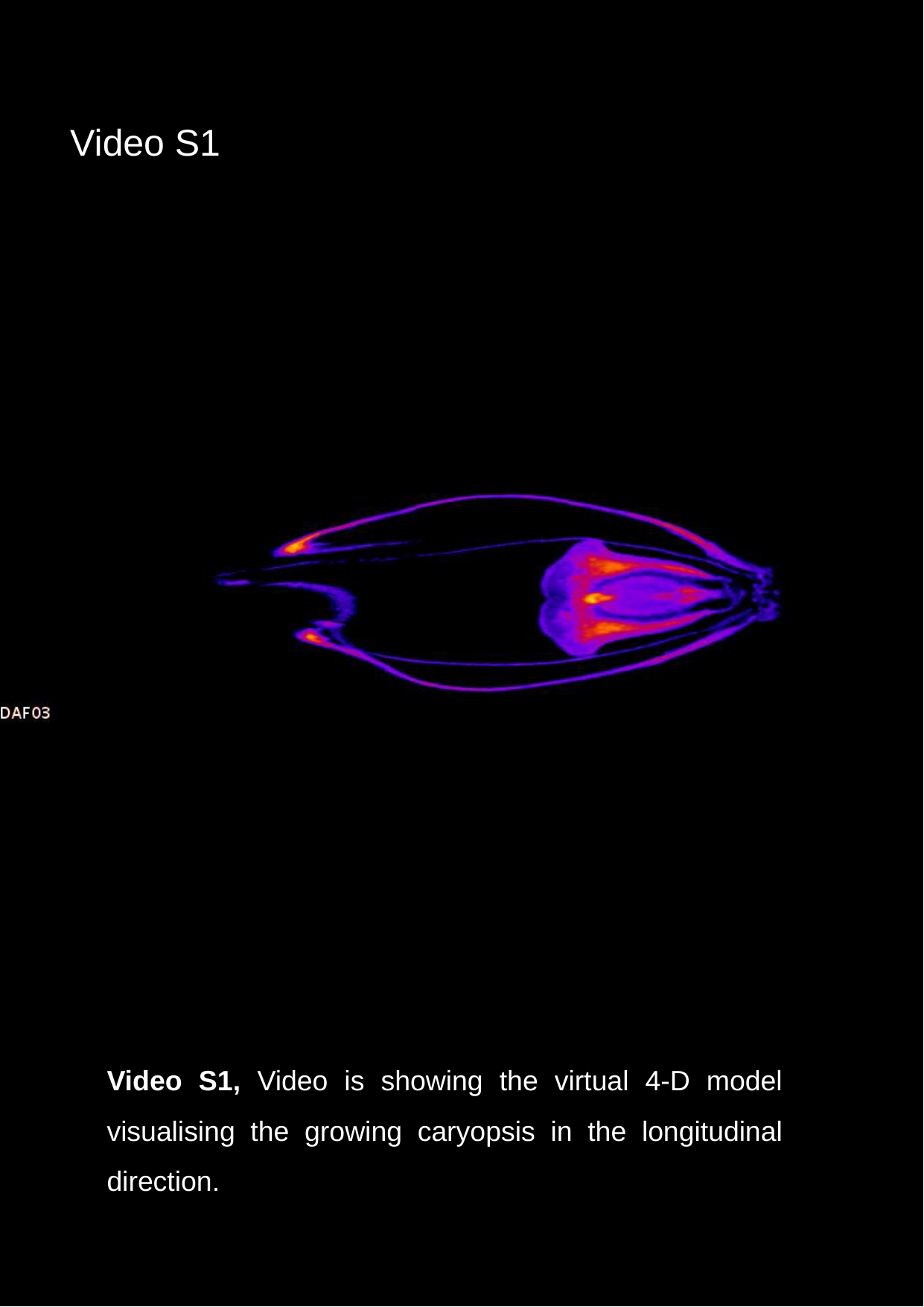

Video S1
Video S1, Video is showing the virtual 4-D model visualising the growing caryopsis in the longitudinal direction.
